# Os–Ru nanozyme with peroxidase-mimicking activity for rapid dual-readout colorimetric analysis of catechins in green tea beverages

**DOI:** 10.1039/d5ra05417f

**Published:** 2025-08-26

**Authors:** Yingying Zhong, Junsong Yang, Qian Liang, Xiao Yu, Hongyu Lai, Yu Lin, Qijie Mo, Qing Wang, Zijian Chen, Hongwu Wang

**Affiliations:** a School of Food & Pharmaceutical Engineering, Zhaoqing University Zhaoqing 526061 People's Republic of China hwwang@zqu.edu.cn; b Laboratory of Quality & Safety Risk Assessment for Agro-products (Zhaoqing), Ministry of Agriculture and Rural Affairs Zhaoqing 526061 People's Republic of China; c Guangdong Engineering Technology Research Center of Food & Agricultural Product Safety Analysis and Testing Zhaoqing 526061 People's Republic of China; d School of Environmental and Chemical Engineering, Zhaoqing University Zhaoqing 526061 People's Republic of China

## Abstract

Catechin, a phenolic-active substance extracted from natural plants, exhibits a wide range of biological activities. However, exceeding safe levels can harm human health, making accurate quantification essential. Current assay methods, however, do not provide efficient or precise catechin measurements. In this study, an Os–Ru nanozyme with outstanding peroxidase-mimicking activity was first developed, showing *K*_m_ values of 0.36 mM for 3,3′,5,5′-tetramethylbenzidine (TMB) and 2.67 mM for H_2_O_2_. Additionally, catechin was found to effectively scavenge hydroxyl radicals (HO˙) generated from H_2_O_2_ decomposition by the Os–Ru nanozyme. Based on this, a rapid dual-readout colorimetric method for catechin detection was established, featuring an absorbance measurement in solution and grayscale (G) analysis on a paper platform, both based on the Os–Ru nanozyme. This dual-readout colorimetric method achieved highly sensitive catechin detection across a broad linear concentration range (0 to 450 μmol L^−1^), with detection limits of 2.84 μmol L^−1^ for absorbance measurement and 9.68 μmol L^−1^ for the *G* value analysis. Moreover, the method demonstrated excellent reliability and accuracy in practical applications, yielding spiked recoveries between 91.78 and 103.01% and relative standard deviations (RSD) ranging from 1.62 to 6.74% in the analysis of green tea beverages. Overall, this Os–Ru nanozyme-based dual-readout colorimetric method has considerable potential for practical catechin detection and could provide additional inspiration and ideas for the rational design and development of colorimetric sensing methods for rapid detection of nanozyme-based antioxidants.

## Abstract 2

A dual-readout colorimetric analysis method based on Os–Ru nanozyme with excellent peroxidase-mimicking activity was developed for catechins rapid detection.

## Graphical abstract

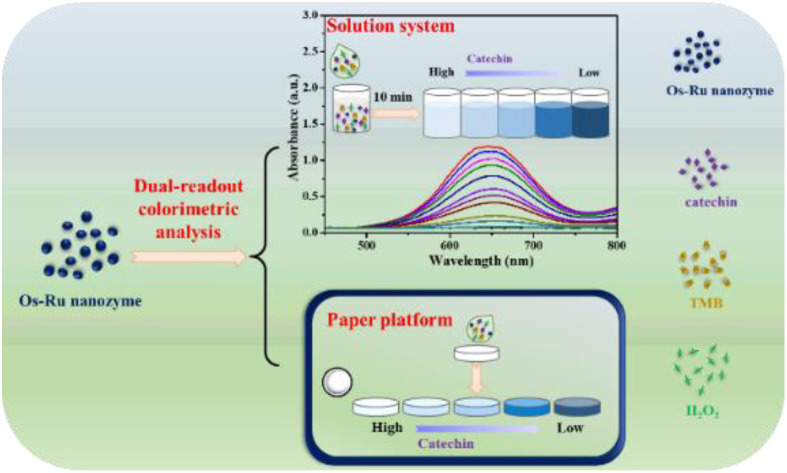

## Introduction

Catechin is a phenolic-active substance extracted from natural plants.^1^ It is commonly found in tea and is the main active ingredient that has biological activity.^2^ Catechin has been reported to possess various bioactivities such as anti-inflammatory, antibacterial, anticancer, cardiovascular protection, blood glucose lowering, and free radical scavenging effects,^3–6^ which has resulted in its widespread use in medical and food applications. However, when catechin levels exceed the safety threshold (800 mg per day), it may cause adverse health effects such as nausea and dizziness.^7^ Therefore, establishing an efficient, sensitive, and reliable catechin detection method is of considerable importance for ensuring food safety.

Currently, gas chromatography (GC), high-performance liquid chromatography (HPLC), Raman spectroscopy, and electrochemical methods are the primary techniques used for catechin determination.^8–11^ However, these approaches often have limitations such as the requirement for large and expensive instrumentation, complex and time-consuming procedures, high costs, and the inability to provide visual results. These drawbacks hinder their ability to meet the current demand for efficient and accurate catechin detection.^12,13^ In contrast, methods like UV spectrophotometry using a microplate reader and paper-based colorimetric assays offer advantages such as a rapid response, simple operation, and relatively low cost.^14,15^ The synthesis of new nanomaterials is essential for the development of sensitive colorimetric sensors for catechin.

Nanozymes are nanomaterials exhibiting enzyme-like activity, as first reported in 2007.^16^ They have gained considerable attention because they overcome the limitations of natural enzymes, such as strict storage requirements, poor stability, and high costs, making them an emerging research focus. Currently, nanozymes are widely applied in bioanalysis, medical therapy, food analysis, and related fields.^17–20^ Notably, nanozymes based on osmium (Os) and ruthenium (Ru) have recently demonstrated unique catalytic properties and promising application potential.^21–24^ For example, Pan *et al.* prepared an Os nanozyme with peroxidase-mimicking activity using a “one-pot method” and applied it in an immunoassay of folic acid.^21^ Zhu *et al.* synthesized a Ru@CeO_2_ nanozyme exhibiting a photothermal effect for rectal cancer treatment by combining chemotherapy with photothermal therapy.^23^ However, research on Os–Ru nanozymes is relatively limited, primarily focusing on applications such as emission cathodes, quantum technologies, and electrocatalysts.^25,26^ To date, no studies have explored their use in food detection.

**Scheme 1:**
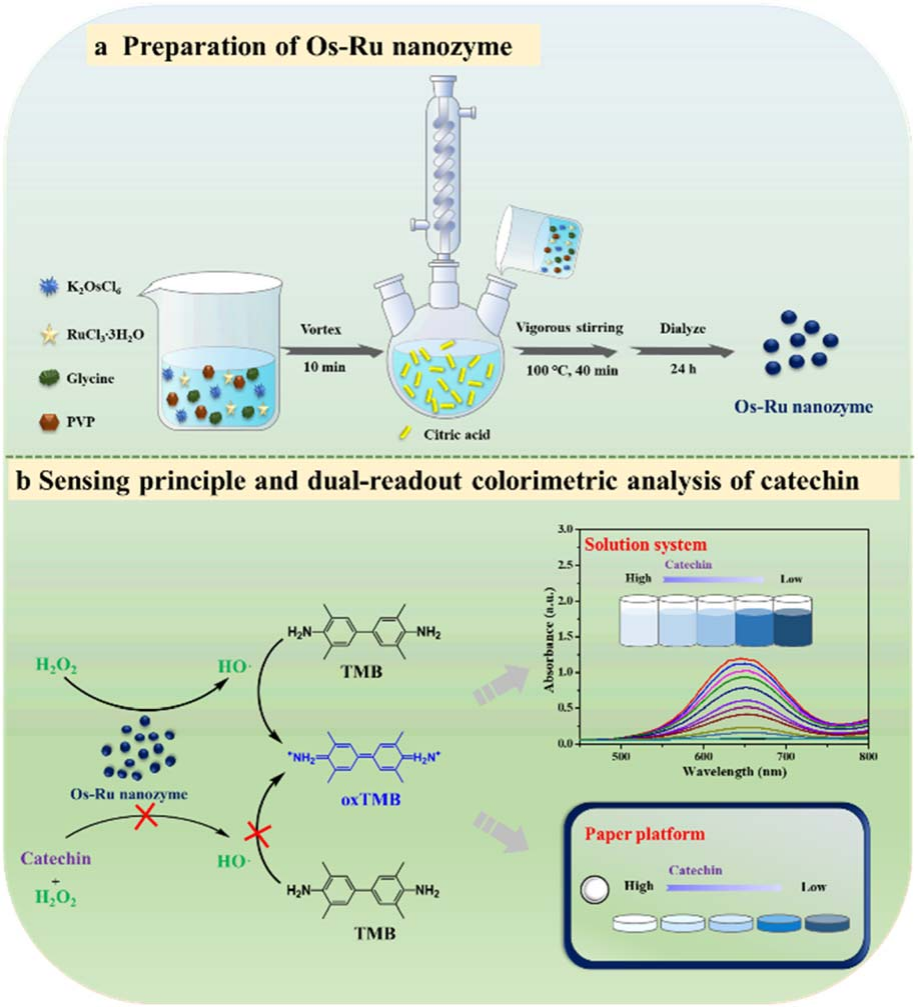
Schematic of (a) Os–Ru nanozyme preparation and (b) sensing principle and dual-readout colorimetric analysis of catechin based on the Os–Ru nanozyme in the solution system and paper platform.

In this study, an Os–Ru nanozyme with excellent peroxidase-mimicking activity was innovatively prepared using a one-step hydrothermal reaction. Based on this nanozyme, rapid dual-readout colorimetric analysis of catechin was achieved, featuring absorbance measurement in solution and grayscale (G) analysis on a paper platform. The sensing principle of this dual-readout colorimetric analysis method is illustrated in Scheme 1. The Os–Ru nanozyme efficiently catalyzes the generation of hydroxyl radicals (HO˙) from H_2_O_2_*via* its peroxidase-mimicking activity, which oxidizes the colorimetric substrate TMB to the blue product oxTMB, causing both the solution and paper platform to turn blue. However, in the presence of catechin, it scavenges the hydroxyl radicals (HO˙) generated by the Os–Ru nanozyme-catalyzed decomposition of H_2_O_2_, thus inhibiting TMB oxidation and resulting in a lighter color for both the solution and paper platform. Consequently, rapid dual-readout colorimetric analysis of catechin can be easily performed based on these color changes. Overall, the nanozyme-based dual-readout colorimetric analysis method developed in this study shows considerable potential for rapid detection of antioxidants such as catechins and offers valuable insights for designing and developing colorimetric sensors.

## Experimental methods

### Preparation of the Os–Ru nanozyme

The Os–Ru nanozyme was synthesized following a previously reported method with slight modifications.^27^ Briefly, 50 μL of 40 mM K_2_OsCl_6_ solution, 50 μL of 20 mM RuCl_3_·3H_2_O solution, 6.0 mg glycine, and 15 mg of PVP were added to a 1 mL tube containing 900 μL of deionized water. After vertexing at 20 °C for 10 min, this mixture was transferred to a three-neck flask containing 50 mL of boiling 3.5 mM citric acid solution. The reaction was then vigorously stirred at 1800 rpm and maintained at 100 °C for 40 min. Finally, the product was placed in a 500-D dialysis bag and dialyzed with deionized water for 24 h to yield the Os–Ru nanozyme stock solution.

### Peroxidase-mimicking activity of the Os–Ru nanozyme

The peroxidase-mimicking activity of the Os–Ru nanozyme was initially assessed by evaluating its ability to catalyze the oxidation of the chromogenic substrate TMB into the blue product oxTMB in the presence of H_2_O_2_. This was followed by a more detailed investigation of its enzyme-mimicking catalytic performance using steady-state kinetic assays.^28^ For complete experimental details, refer to the “peroxidase-mimicking activity of the Os–Ru nanozyme” section in the SI.

### Mechanism of colorimetric analysis of catechin based on the Os–Ru nanozyme

The mechanism of colorimetric analysis of catechin using the Os–Ru nanozyme was explored by tracking the production of hydroxyl radicals (HO˙) in the reaction system. Terephthalic acid (TA) and 5,5-dimethyl-1-pyrroline-*N*-oxide (DMPO) were employed as HO˙ scavengers. Initially, the fluorescence spectra of the reaction mixture (TA (5 mM, 100 μL), H_2_O_2_ (10 mM, 800 μL), and the Os–Ru nanozyme (64-fold dilution of the original solution, 50 μL)) were recorded with and without catechin using a fluorescence spectrophotometer at 315 nm excitation. Before measurement, the reaction systems were incubated at 45 °C for 30 min to allow the reaction to proceed. Moreover, the electron spin resonance (ESR) spectra of the reaction system (DMPO (50 mM, 100 μL), the Os–Ru nanozyme (64-fold dilution of the original solution, 50 μL), H_2_O_2_ (10 mM, 800 μL)) were recorded with and without catechin using a Bruker A300 spectrometer. Before measurement, the reaction mixtures were stirred for 5 min at pH 6.5.

### Anti-interference ability of dual-readout colorimetric detection of catechin

The anti-interference ability of the Os–Ru nanozyme-based dual-readout colorimetric assay for catechin was assessed by examining the inhibitory effects of common food interferents (such as ions, sugars, and amino acids) on the reaction system (Os–Ru + TMB + H_2_O_2_).^29^ A concentration of 900 μmol L^−1^ was selected for each interfering substance, tested both in solution and on a paper platform. The detailed experimental procedures were as follows.

In the solution system (absorbance measurement), 20 μL of Os–Ru nanozyme solution (256-fold dilution) of the original solution, 10 μL of catechin solution (450 μmol L^−1^), and/or 10 μL of interfering substance solution (900 μmol L^−1^), along with 200 μL of reaction substrate solution (containing 2.0 mM TMB and 10 mM H_2_O_2_), were first mixed in 96-well plates. The mixture was then incubated at 20 °C and pH 4.5 for 10 min. Then, the absorbance was measured at 650 nm using a microplate reader. A blank control group, without catechin and interfering substances, was included for comparison.

On the paper platform (*G* value analysis), 10 μL of the Os–Ru nanozyme solution (64-fold dilution of the original solution) was first dropwise added to clean test paper with an 8 mm diameter. After drying naturally, the catechin detection paper was prepared. Next, 10 μL of catechin solution (450 μmol L^−1^) and/or 10 μL of interfering substances solution (900 μmol L^−1^) were added dropwise to the test paper and allowed to dry naturally at 20 °C. Then, 20 μL of reaction substrate solution (containing 3.75 mM TMB and 10 mM H_2_O_2_) was added dropwise, followed by a 10 min reaction at 20 °C and pH 4.0. Next, images of the test papers were captured using the device shown in Fig. S1. The device consisted of an iPhone 14 Pro and a small LED photography studio. During image capture, the iPhone 14 Pro was kept fixed at the top of the photography studio, and the LED lights in the studio were kept on to minimize variability caused by image acquisition conditions. The *G* value of the test paper was obtained by analyzing the images with ImageJ software. The group without catechin, interfering substances, or reaction substrate served as the control group, while the group without catechin and interfering substances was designated as the blank group. Δ*G* was defined as the difference between the *G* value of the control group and that of the test group.

### Dual-readout colorimetric detection of catechin based on the Os–Ru nanozyme

Briefly, in the solution system (absorbance value readout), the Os–Ru nanozyme solution (20 μL, diluted 256-fold dilution of the original solution), catechin solutions at varying concentrations (0 to 450 μmol L^−1^, 10 μL), and reaction substrate solution (200 μL, containing 2.0 mM TMB and 10 mM H_2_O_2_) were first mixed in 96-well plates. The mixture was then incubated for 10 min at 20 °C and pH 4.5. Then, the absorbance of the system at 650 nm was measured using a microplate reader. The group without reaction substrate served as the blank control, and all final absorbance values were obtained by subtracting the blank control absorbance from the measured values. Finally, the absorbance values were plotted as the vertical axis, and the catechin concentrations were plotted as the horizontal axis.

The limit of detection (LOD) of the solution system for catechin analysis was calculated according to eqn (1) when the signal-to-noise ratio (S/N) was 3.

1 LOD = 3 × SD/*m*,

where SD is the standard deviation of the signal measured multiple times in the blank control group, and *m* represents the slope of the standard curve.

On the paper platform (*G* value analysis), catechin solutions at varying concentrations (0 to 450 μmol L^−1^, 10 μL) were first added dropwise to the test paper. After air-drying at 20 °C, the reaction solution (20 μL, containing 3.75 mM TMB and 10 mM H_2_O_2_) was applied dropwise. The test paper was then incubated at 20 °C and pH 4.0 for 10 min, followed by photographing with a device consisted of an iPhone 14 Pro and a small LED photography studio. The *G* value of the test paper was obtained by analyzing the photos using ImageJ software. The *G* values were plotted on the vertical axis against catechin concentrations on the horizontal axis. The subsequent procedures were consistent with those described for the solution system above.

### Dual-readout colorimetric analysis of catechin in real samples

To verify the reliability and application potential of the Os–Ru nanozyme for detecting catechin in real samples, we selected three types of green tea beverages (Master Kong, Vita, and Yulu) for spiked recovery analysis first. The pretreatment and spiked recovery procedures followed literature methods with minor modifications.^27^ First, the green tea beverages were centrifuged at 12 000 rpm and 20 °C for 10 min to collect the supernatant. Next, the supernatants were diluted 100-fold with distilled water and filtered through 0.22 μm filters. Then, varying amounts of catechin were weighed and added to the dilutions of the three beverages to prepare spiked recovery solutions with concentrations of 400, 200, and 0 μmol L^−1^. The catechin contents in these spiked beverages were then measured both in the solution and on the paper platform. Then, the spiked recovery rates and relative standard deviations (RSD) were calculated. Additionally, high-performance liquid chromatography (HPLC) was performed for validation of the results.

Finally, Master Kong was selected as an example to measure the catechin content in real green tea beverages. The pretreatment (centrifugation, dilution and filtration) and measurement steps were consistent with those described above.

## Results and discussion

### Characterization of the Os–Ru nanozyme

**Fig. 1:**
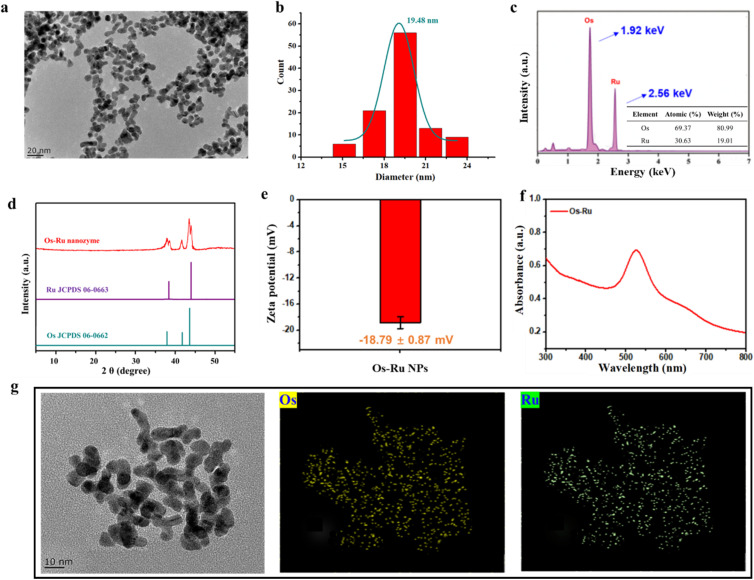
(a) TEM image, (b) size distribution, (c) EDS spectrum (inset was the ratio of Os to Ru elements), (d) XRD patterns, (e) zeta potential, (f) UV-vis spectra and (g) EDS mapping images of the elements Os and Ru of the Os–Ru nanozyme.

To confirm the successful synthesis of the Os–Ru nanozyme, we performed TEM, EDS, ICP-MS, XRD, EDS mapping, zeta potential, and UV-vis spectroscopy. Fig. 1a and b show that the Os–Ru nanozyme consists of dispersed, irregularly shaped particles with an average size of approximately 19.48 nm. Additionally, the EDS elemental analysis shown in Fig. 1c indicates that the nanozyme contains only Os and Ru, with characteristic EDS peaks at 1.91 and 2.56 keV, and atomic ratios of 69.37% and 30.63%, corresponding to Os and Ru, respectively. Moreover, the contents of Os and Ru elements in the Os–Ru nanozyme were measured by ICP-MS, and the results were calculated as 69.42% and 30.58% for Os and Ru, respectively, consistent with the aforementioned EDS analysis results. Furthermore, the XRD results (Fig. 1d) show that the Os–Ru nanozyme exhibits five diffraction peaks at 38.0, 38.4, 41.8, 43.6, and 44.0°, which correspond to the (100), (002), and (101) planes of fcc Os (JCPDS 06-0662) and the (100) and (101) planes of fcc Ru (JCPDS 06-0663), suggesting that the Os–Ru nanozyme was alloy structure. Fig. 1g EDS mapping result showed that both Os and Ru elements were uniformly distributed throughout the Os–Ru nanozyme, further confirming its alloy structure. Additionally, Fig. 1e and f show that the zeta potential of the Os–Ru nanozyme is approximately −18.79 ± 0.87 mV, with a maximum absorption peak at approximately 526 nm. These findings confirm the successful synthesis of the Os–Ru nanozyme.

### Peroxidase-mimicking activity of the Os–Ru nanozyme

To verify whether Os–Ru nanozyme exhibit peroxidase-mimicking activity, TMB was chosen as the chromogenic substrate to test if it could be oxidized to the blue product oxTMB in the presence of H_2_O_2_. As shown in Fig. S2, Os–Ru NPs alone could not directly oxidize TMB, indicating they lack oxidase-mimicking activity. However, upon adding H_2_O_2_ to the system, the solution turned blue and showed a strong absorption peak at approximately 650 nm. This demonstrated that Os–Ru NPs can catalyze the oxidation of TMB to oxTMB in the presence of H_2_O_2_, confirming their peroxidase-mimicking activity.

To further evaluate the peroxidase-mimicking activity of Os–Ru NPs, we performed a steady-state kinetic study. The Michaelis–Menten constant (*K*_m_) indicates the enzyme's affinity for a substrate, with a lower *K*_m_ representing stronger affinity. The maximum initial velocity (*V*_max_) represents the fastest rate of the catalytic reaction, where a higher value means a quicker reaction. According to Fig. S3 and Table 1, Os–Ru NPs showed *K*_m_ values of 0.36 mM for TMB and 2.67 mM for H_2_O_2_, while their *V*_max_ values were 337.29 × 10^−8^ M s^−1^ for TMB and 208.37 × 10^−8^ M s^−1^ for H_2_O_2_. Moreover, as shown in Table 1, the Os–Ru NPs demonstrated higher affinity and faster reaction rates toward both TMB and H_2_O_2_ compared to horseradish peroxidase (HRP) and many other nanozymes, indicating that the prepared Os–Ru NPs possess excellent peroxidase-mimicking activity and are promising candidates as nano-peroxidases.

**Table 1:** Comparison of *K*_m_ and *V*_max_ between the Os–Ru nanozyme and other catalysts with TMB or H_2_O_2_ as substrates

Catalyst	TMB		H_2_O_2_		Ref.
	*K*_m_ (mM)	*V*_max_ (10^−8^ M s^−1^)	*K*_m_ (mM)	*V*_max_ (10^−8^ M s^−1^)	
HRP	0.43	10.00	3.70	8.71	30
CuCeTA nanoflowers	0.54	0.14	0.62	1.92	31
SACe–N–C nanowires	0.11	90.16	33.16	225.06	32
PdCu_0.9_ nanoalloys	—	—	57.33	5.61	33
PB nanoparticles	0.41	196.17	23.20	198.17	34
Fe–N–C SAzymes	3.60	116.00	12.20	35.60	35
Pt clusters	2.90	69.00	49.10	85.00	36
GeO_2_ nanoparticles	0.42	23.30	1.75	23.40	37
Os–Ru nanozyme	0.36	337.29	2.67	208.37	This work

### Long-term stability of the Os–Ru nanozyme

To assess the practical application potential of the Os–Ru nanozyme, the long-term stability of its structure and peroxidase-mimicking activity were evaluated. As shown in Fig. S4, after 90 days of storage at 20 °C in sealed centrifuge tubes, the Os–Ru nanozyme retained its original morphology, appearing as dispersed irregularly shaped particles (Fig. S4a), maintained a consistent size of approximately 19.32 nm (Fig. S4b). Moreover, it exhibited a same XRD pattern as before storage (Fig. S4c) and nearly unchanged peroxidase-mimicking activity after storage (Fig. S4d). These findings suggest that the Os–Ru nanozyme has excellent long-term stability in both structure and peroxidase-mimicking activity.

### Feasibility of dual-readout colorimetric detection of catechin based on the Os–Ru nanozyme

**Fig. 2:**
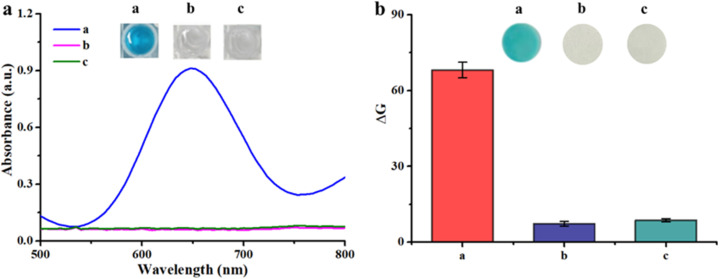
Feasibility of using the Os–Ru nanozyme for dual-readout colorimetric detection of catechin (a) in solution and (b) on the paper platform. (a: Os–Ru nanozyme + TMB + H_2_O_2_, b: Os–Ru nanozyme + TMB + H_2_O_2_ + catechin (450 μmol L^−1^), c: TMB + H_2_O_2_).

To evaluate the feasibility of using the Os–Ru nanozyme for dual-readout colorimetric detection of catechin (absorbance measurement in solution and *G* value analysis on a paper platform), the impact of catechin on the color, absorbance, and *G* value of the system (Os–Ru nanozyme + TMB + H_2_O_2_) was investigated in both platforms. As shown in Fig. 2, when only TMB, H_2_O_2_, and the Os–Ru nanozyme were present, the solution (Fig. 2a, well a) and paper platform (Fig. 2b, paper a) both displayed a blue color, corresponding to a high absorbance value at 650 nm and a Δ*G* value (*G* value of the blank group minus *G* value of the sample group). These phenomena result from the Os–Ru nanozyme catalyzes H_2_O_2_ to oxidize TMB to produce blue oxTMB. However, when a high concentration of catechin (450 μmol L^−1^) was introduced to both the solution and paper platforms, the color changed from blue to colorless in the solution (Fig. 2a, well b) and on the paper (Fig. 2b, paper b), accompanied by a considerable decrease in absorbance at 650 nm and Δ*G* value. These results demonstrate that catechin effectively inhibits the oxidation of TMB in system (Os–Ru nanozyme + TMB + H_2_O_2_) both in solution and on paper platform. Thus, the Os–Ru nanozyme can be employed to develop dual-readout colorimetric detection platforms for catechins in solution and on paper.

### Mechanism of colorimetric detection of catechin based on the Os–Ru nanozyme

Studies have shown that nanozymes exhibit peroxidase-mimicking activity by catalyzing the decomposition of H_2_O_2_ into hydroxyl radicals (HO˙), which then oxidize chromogenic substrates such as TMB and ABTS.^38^ Based on this mechanism, it is reasonable to speculate that catechin inhibits TMB oxidation by scavenging the HO˙ produced during the Os–Ru nanozyme-catalyzed decomposition of H_2_O_2_. To verify this hypothesis, terephthalic acid (TA) and 5,5-dimethyl-1-pyrroline-*N*-oxide (DMPO) were used as HO˙ trapping agents. The amount of HO˙ generated in the presence or absence of catechin was then monitored using fluorescence spectroscopy and electron spin resonance (ESR) spectroscopy.

**Fig. 3:**
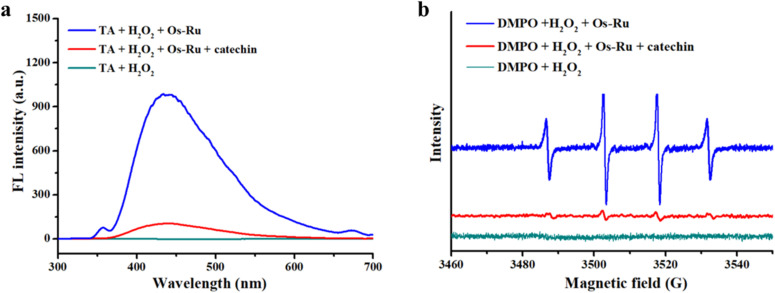
Mechanism of colorimetric detection of catechin based on the Os–Ru nanozyme. (a) Fluorescence spectra of the TA + H_2_O_2_ + Os–Ru system with or without catechin (450 μmol L^−1^). (b) ESR spectra of the DMPO + Os–Ru + H_2_O_2_ system with or without catechin (450 μmol L^−1^).

The results in Fig. 3a showed that the system containing only TA and H_2_O_2_ had no fluorescence emission peak at approximately 435 nm, while the system containing both TA, H_2_O_2_ and Os–Ru nanozyme exhibited a strong fluorescence emission peak at approximately 435 nm, indicating that H_2_O_2_ produced a large amount of HO˙ catalyzed by Os–Ru nanozyme. These HO˙ was captured by TA to form the fluorescent TA–OH product. However, when catechin (450 μmol L^−1^) was added to this system, the fluorescence intensity considerably decreased, implying that catechin effectively scavenged the HO˙ produced by the Os–Ru nanozyme-catalyzed H_2_O_2_. Similarly, as shown in Fig. 3b, the system containing only DMPO and H_2_O_2_ did not show ESR signal, whereas the system containing both DMPO, H_2_O_2_ and Os–Ru nanozyme produced a strong ESR signal at a 1 : 2 : 2 : 1 ratio, characteristic of HO˙ radicals, which further suggested that Os–Ru nanozyme catalyzed the production of HO˙ from H_2_O_2_. When catechin (450 μmol L^−1^) was added to this system, the ESR signal was nearly absent, further confirming catechin's ability to scavenge HO˙ generated by the Os–Ru nanozyme-catalyzed decomposition of H_2_O_2_.

### Optimization of Os–Ru nanozyme sensing conditions

To achieve optimal sensing performance of the Os–Ru nanozyme for dual-readout colorimetric analysis (absorbance measurement in solution and *G* value analysis on a paper platform), we carefully optimized the sensing conditions for both the solution system and the paper platform. The results are presented in Fig. S5 and S6.

For the solution system (absorbance measurement), the optimizable conditions included the Os–Ru nanozyme concentration (expressed as the dilution ratio), reaction time, pH of the reaction system, and reaction temperature. The optimal sensing conditions were selected where the absorbance at 650 nm ranged from 0.8 to 1.2.^39^ Following optimization, the ideal parameters were a dilution ratio of 256 for the Os–Ru nanozyme, a reaction time of 10 min, a pH of 4.5, and a temperature of 20 °C. For a detailed analysis and discussion, please refer to the SI.

For the paper platform (*G* value analysis), the optimizable conditions included the Os–Ru nanozyme concentration (expressed as dilution ratio), reaction time, reaction system pH, and TMB concentration. After optimization, the ideal sensing parameters for the paper platform included a dilution ratio of 64 for the Os–Ru nanozyme, a reaction time of 10 min, a pH of 4.0, and a TMB concentration of 3.75 mM. For detailed analysis and discussion, please refer to the SI.

### Anti-interference capability of dual-readout colorimetric detection of catechin

To evaluate the anti-interference capability of the dual-readout colorimetric analysis of catechin based on the Os–Ru nanozyme, common ions, sugars, and amino acids typically found in food were chosen as potential interfering substances. The impact of these substances on the reaction system (Os–Ru + TMB + H_2_O_2_) was examined both in solution and on the paper platform. Additionally, whether catechin could still effectively inhibit the color change of the reaction system in the presence of these interfering substances was also assessed.

**Fig. 4:**
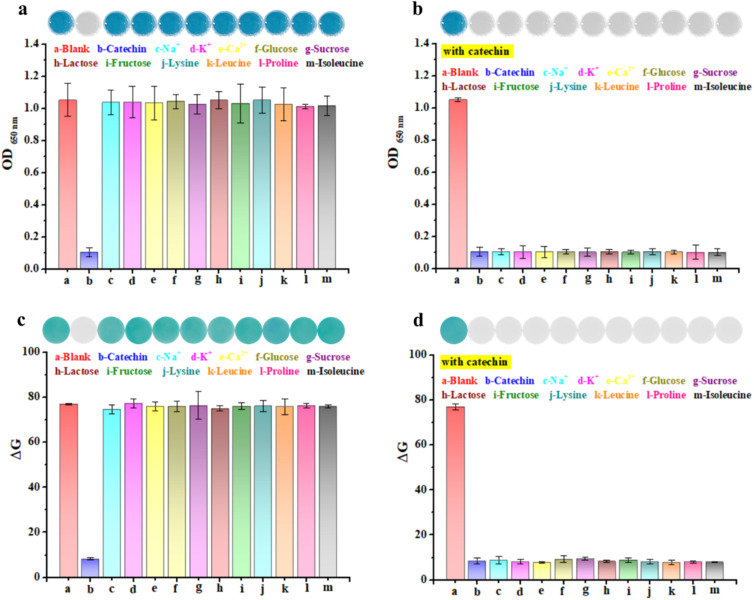
Anti-interference ability of dual-readout colorimetric detection of catechin. (a and b) Color and absorbance at 650 nm of the Os–Ru nanozyme + H_2_O_2_ + TMB system with catechin or/and interfering substances in the solution. (c and d) Color and gray (G) scale value of the Os–Ru nanozyme + H_2_O_2_ + TMB system with catechin and/or interfering substances on the paper platform.

Fig. 4a and c indicate that under identical conditions, both the sample wells and paper with catechin remained colorless, corresponding to very low absorbance values at 650 nm and low Δ*G* values. In contrast, the blank control well (paper) and the sample wells (papers) containing only the interfering substances all showed a blue color, exhibiting similar absorbance and Δ*G* values. Furthermore, Fig. 4b and d show that when both catechin and interfering substances were present, all wells and papers (except the blank control group) remained colorless, with low absorbance and Δ*G* values. These findings indicate that the Os–Ru nanozyme used for the dual-readout colorimetric detection of catechin has strong anti-interference capability.

### Dual-readout colorimetric detection of catechin using the Os–Ru nanozyme

**Fig. 5:**
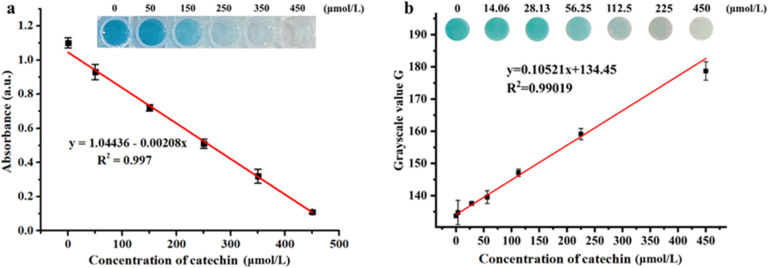
Sensitivity and linear range of the dual-readout colorimetric method for catechin detection. Color changes and linear curves of (a) the solution system and (b) the paper platform.

Based on the previously demonstrated excellent anti-interference capability, the sensitivity and linear range of the dual-readout colorimetric detection for catechin using the Os–Ru nanozyme were assessed. This dual-readout colorimetric detection includes absorbance measurement in solution and *G* value analysis on a paper platform. As shown in Fig. 5, increasing catechin concentration gradually lightened the color of both the solution (Fig. 5a) and the paper (Fig. 5b) from the original blue to colorless. This effect occurs because catechin effectively scavenges hydroxyl radicals (HO˙) generated in the system. Consequently, higher catechin concentrations reduce the amount of residual HO˙, inhibiting the oxidation of TMB. Moreover, Fig. 5 shows that catechin exhibited good linear relationships for both the solution absorbance values (Fig. 5a) and the paper *G* values (derived by converting paper color using ImageJ software, Fig. 5b) across the concentration range of 0 to 450 μmol L^−1^, with correlation coefficients (*R*^2^) of 0.997 and 0.990, respectively. The limits of detection (LOD) were calculated to be 2.84 μmol L^−1^ for the solution system (absorbance measurement) and 9.68 μmol L^−1^ for the paper platform (*G* value analysis), with both lower and thus outperforming many previously reported methods (Table 2). Additionally, the results in Table 2 also show that the time (10 min) required for catechin dual-read colorimetric detection using Os–Ru nanozyme is significantly shorter than many previously reported methods. In summary, the dual-readout colorimetric method based on the Os–Ru nanozyme demonstrates high sensitivity, wide linear range and short detection time for catechin detection.

**Table 2:** Comparison of catechin detection using different assays

Material	Sensing modes	Linear range (μmol L^−1^)	LOD (μmol L^−1^)	Detection time (min)	Ref.
Papain	Colorimetric	344–2752	1.5 × 10^3^	60	40
CuAsp nanofibers	Colorimetric	20–1200	5.89	20	9
ZnONPs	Fluorescence	34.45–413.41	—	—	41
MI–Bpy–Cu nanozyme	Colorimetric	0–344.5	2.86	30	29
Triaminotriazine-based polyimide (PI) film	Electrochemistry	50–350	15.2	—	42
Tyrosinase & MBTH	Paper colorimetric	80–1000	71	13	43
Os–Ru nanozyme	Colorimetric	0–450	2.84	10	This work
Os–Ru nanozyme	Paper colorimetric	0–450	9.68	10	This work

In addition, the stability of the paper platform after 60 days of storage at room temperature (20 °C) was assessed. As shown in Fig. S7, the paper platform showed no considerable loss of activity over this period, demonstrating excellent storage stability at room temperature. This supports its practical application for catechin testing.

### Dual-readout colorimetric analysis of catechin in real samples

**Fig. 6:**
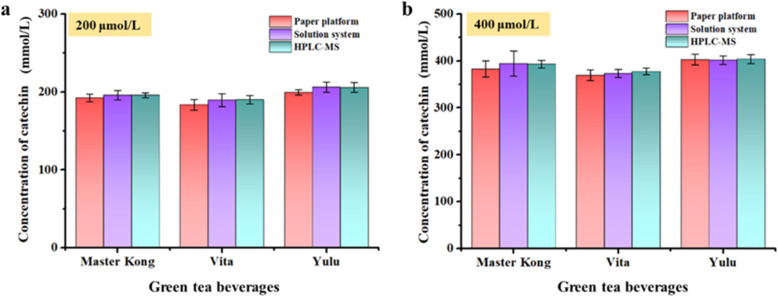
Spiked real sample test results obtained using the dual-readout colorimetric analysis method using the Os–Ru nanozyme and HPLC for comparison. Spiked concentrations were 200 μmol L^−1^ (a) and 400 μmol L^−1^ (b).

To evaluate the feasibility and reliability of the Os–Ru nanozyme for dual-readout colorimetric detection of catechin in real samples, three green tea beverages containing catechin were selected for spiked recovery tests first. The results were compared with those obtained by HPLC. As shown in Fig. 6, the catechin spiked recovery concentrations measured both in the solution and on the paper platform closely matched those obtained by HPLC. Moreover, as shown in Table S1, the catechin spiked recoveries measured in the solution system and on the paper platform ranged from 93.27 to 103.01% and from 91.78 to 100.76%, respectively, with relative standard deviations (RSD) of 2.17 to 6.74% and 1.62 to 4.57%, respectively. These values closely align with the catechin spiked recoveries (94.36 to 102.83%) and RSD (1.69 to 2.97%) obtained *via* the HPLC method. These findings show that the dual-readout colorimetric method based on the Os–Ru nanozyme exhibits excellent accuracy and reliability for detecting catechin in real samples.

Based on this, the catechin content in real green tea beverages (using Master Kong as example) was measured finally. The results in Table S2 showed that the catechin content in Master Kong green tea beverage measured through the dual-readout colorimetric analysis method were 2208.96 μmol L^−1^ (in the solution system) and 2178.68 μmol L^−1^ (on the paper platform), respectively.

## Conclusions

The Os–Ru nanozyme was successfully synthesized for the first time *via* a one-step hydrothermal reaction in this study and exhibited outstanding peroxidase-mimicking activity. The *K*_m_ values for substrates TMB and H_2_O_2_ were 0.36 and 2.67 mM, respectively, which are lower than those of HRP and many other nanozymes, highlighting its potential as a superior nano-peroxidase candidate. Furthermore, it was demonstrated that catechin effectively scavenges HO˙ generated during the decomposition of H_2_O_2_ catalyzed by the Os–Ru nanozyme, thus inhibiting the oxidation of the colorimetric substrate TMB in the reaction system. Based on this, a dual-readout rapid colorimetric method for catechin detection was developed by optimizing the Os–Ru nanozyme sensing conditions. This method combines absorbance measurement in solution with *G* value analysis on a paper platform, offering high sensitivity and a wide linear detection range of 0 to 450 μmol L^−1^. The detection limits were 2.84 μmol L^−1^ for absorbance and 9.68 μmol L^−1^ for the *G* value, both lower than many existing methods. Additionally, this dual-readout method showed excellent accuracy and reliability in real sample analysis, with spiked recoveries ranging from 91.78 to 103.01% and RSD values between 1.62 and 6.74%, indicating considerable potential for practical applications. Overall, this study offers valuable insights for the rational design and development of nanozyme-based colorimetric sensing methods for rapid antioxidant detection, thus expanding the practical, fast, and on-site application of nanozymes in antioxidant monitoring.

## Author contributions

Yingying Zhong: contributed to conceptualization, methodology, validation, formal analysis, investigation, data curation, writing – original draft and funding acquisition. Junsong Yang: contributed to methodology, formal analysis, investigation, data curation and writing – original draft. Qian Liang: contributed to methodology, data curation, writing and funding acquisition. Xiao Yu: contributed to methodology, data curation, writing and funding acquisition. Hongyu Lai: contributed to writing and editing. Yu Lin: contributed to writing and editing. Qijie Mo: contributed to writing – review, supervision and funding acquisition. Qing Wang: contributed to writing – review, supervision and funding acquisition. Zijian Chen: contributed to writing – review and supervision. Hongwu Wang: contributed to writing – review, supervision and funding acquisition.

## Conflicts of interest

The authors have read the policy on conflicts of interest and declare no competing interests.

## Supplementary Material

RA-015-D5RA05417F-s001

## Data Availability

All underlying data are available in the article itself and its SI. The raw data are available on request from the corresponding author. See DOI: https://doi.org/10.1039/d5ra05417f.
