# Supplementary material for: Os–Ru nanozyme with peroxidase-mimicking activity for rapid dual-readout colorimetric analysis of catechins in green tea beverages
Source: RSC Adv. 2025 Aug 26;15(37):30446–55. doi: 10.1039/d5ra05417f (PMC12380047; doi:10.1039/d5ra05417f)
Supplement: RA-015-D5RA05417F-s001 [file RA-015-D5RA05417F-s001.pdf]

Supplementary Information (SI) for RSC Advances.
This journal is © The Royal Society of Chemistry 2025

Supporting Information

Os–Ru nanozyme with peroxidase-mimicking activity for rapid dual-readout colorimetric analysis of catechins in green tea beverages

Yingying Zhong,[#a,b,c] Junsong Yang,[#d] Qian Liang,[a] Xiao Yu,[a] Hongyu Lai,[a] Yu Lin,[a] Qijie Mo,[a,b,c] Qing Wang,[a,b,c] Zijian Chen,[a,b,c] and Hongwu Wang[*a,b,c]

a. School of Food & Pharmaceutical Engineering, Zhaoqing University, Zhaoqing, 526061, People's Republic of China;

b. Laboratory of Quality & Safety Risk Assessment for Agro-products (Zhaoqing), Ministry of Agriculture and Rural Affairs, Zhaoqing, 526061, People's Republic of China;

c. Guangdong Engineering Technology Research Center of Food & Agricultural Product Safety Analysis and Testing, Zhaoqing, 526061, People's Republic of China;

d. School of Environmental and Chemical Engineering, Zhaoqing University, Zhaoqing, 526061, People's Republic of China

***Corresponding Author:**

Prof. Hongwu Wang

E-mail address: hwwang@zqu.edu.cn

Experimental section

Materials and instruments

Potassium hexachloroosmate (K_2OsCl_6), ruthenium chloride trihydrate ($RuCl_3·3H_2O$), glycine, polyvinylpyrrolidone (PVP, Mw = 24000), ascorbic acid, 3,3',5,5'-tetramethylbenzidine (TMB) and hydrogen peroxide (H_2O_2) were purchased from Aladdin Ltd. (Shanghai, China). Catechin, citric acid monohydrate, sodium phosphate dibasic, dimethyl sulfoxide (DMSO), potassium chloride (KCl), calcium chloride ($CaCl_2$), sodium chloride (NaCl), glucose, sucrose, lactose, fructose, lysine, leucine, proline and isoleucine were offered by Sigma Aldrich (Shanghai, China). Terephthalic acid (TA), 2,2-dimethyl-3,4-dihydro-2H-pyrrole 1-oxide (DMPO), deionized water, test paper and other reagents were received from Macklin (Shanghai, China). Green tee beverages were purchased from local market.

The morphology and elemental analysis of Os–Ru nanozyme were monitored by Transmission electron microscope (TEM, HT7700, Hitachi, Japan). The content of Os and Ru elements in Os–Ru nanozyme was evaluated by Inductively coupled plasma mass spectrometry (ICP-MS, ICAP RQ, Thermo Fisher, Germany). The X-ray diffractogram of Os–Ru nanozyme was gained via X-ray diffraction (XRD, D8 ADVANCE, Bruker, Germany). Zeta potential was determined by Zeta sizer Nano ZS (Malvern, UK). Microplate reader (Infinite 200, Tecan, Austria) was used for measuring the UV absorption spectra and absorbance of the reaction system, and explore the steady-state kinetic of Os–Ru nanozyme. Fluorescence spectrophotometer (Fluorolog-3, HORIBA Instruments Incorporated, USA) and Bruker A300 spectrometer (Bruker, Germany) were utilized to monitor the generation of HO·. A device consisted of an iPhone 14 Pro and a small LED photography studio (**Fig. S1**) was used for capturing images of the test papers. The ImageJ software was used to measure the average size of Os–Ru nanozyme and the grayscale values of test papers.

Peroxidase-mimicking activity of the Os–Ru nanozyme

The peroxidase-mimicking activity of Os–Ru nanozyme was first evaluated through whether it could catalyze the oxidation of the chromogenic substrate TMB in the presence of H_2O_2. Briefly, Os–Ru nanozyme (256-fold dilution of the original solution, 20 μL) and reaction substrate solution (200 μL), which consisted of TMB (2.0 mM) and H_2O_2 (10 mM), were added in 96-well plate and reacted at 20°C for 10 min. After that, the UV-vis absorption curve (500~800 nm) was recorded by microplate reader. A control groups (without Os–Ru nanozyme or H_2O_2) were set in the test to observe the effect of H_2O_2 or Os–Ru nanozyme oxidizing TMB. A blank group (without H_2O_2 and TMB) was set in the test, and all results obtained were removed the blank group value.

The catalytic property of Os–Ru nanozyme was further explored by steady-state kinetic assay. In short, Os–Ru nanozyme (256-fold dilution of the original solution, 20 μL) was mixed with reaction substrate solution (200 μL) in 96-well plate, which consisted of H_2O_2 (10 mM) and different concentration of TMB (2.0, 1.0, 0.5, 0.25, 0.125, 0.0625 mM), or TMB (2.0 mM) and different concentration of H_2O_2 (10, 5, 2.5, 1.25, 0.625, 0.3125, 0.15625 mM). After reacting at 20°C for 10 min, the absorbance of system at 650 nm was measured using microplate reader. The group without TMB and H_2O_2 was set as blank control, and all results obtained were removed the absorbance value of blank group. The concentration of oxTMB produced by the above system was calculated according to the following equation:

$$A = \xi bc \qquad (1)$$

Where A represents the absorbance value of the system measured at 650 nm, ξ is the molar absorption coefficient of oxTMB (3.9×10^4/M·cm),[1] b is the thickness of the absorption layer, and c represents the concentration of oxTMB.

Then the concentration of oxTMB produced per unit time (V_0) was gained on based on the following formula:

$$V_0 = \frac{c}{t} \qquad (2)$$

Where t represents the reaction time, and it is 10 min in this assay.

Next, Lineweaver-Burk plots were draw with the reciprocal of the oxTMB concentration produced per unit time ($\frac{1}{V_0}$) as the vertical axis and the reciprocal of the substrate TMB (or H_2O_2) concentration ($\frac{1}{[S]}$) as the horizontal axis. Finally, the maximum reaction velocity (V_{max}) and the Michaelis-Menten constant (K_m) were calculated using the following formula:

$$\frac{1}{V_0} = \frac{K_m}{V_{max}} \left(\frac{1}{[S]} + \frac{1}{K_m}\right) \qquad (3)$$

Long-term stability of the Os–Ru nanozyme

The long-term stability of the Os–Ru nanozyme was evaluated through monitoring its structure and peroxidase-mimicking activity changes after storage for 90 days. The specific storage conditions were as follows: the Os–Ru nanozyme original solution was placed in centrifuge tubes, sealed with sealing film, and stored in an air-conditioned room at 20°C. Additionally, to reflect actual field conditions, no specific settings were imposed on light, oxygen, or humidity during storage, which were allowed to vary naturally with weather conditions. Briefly, Os–Ru nanozyme (256-fold dilution of the original solution, 20 μL) and reaction substrate solution (200 μL), which consisted of TMB (2.0 mM) and H_2O_2 (10 mM), were mixed in 96-well plate and reacted at 20°C for 10 min. Then, the absorbance value of the system at 650 nm was measured using microplate reader. The absorbance value measured at day 0 was set to 100% peroxidase-mimicking activity.

Feasibility of dual-readout colorimetric detection of catechin based on the Os–Ru nanozyme

The feasibility of Os–Ru nanozyme used for dual-readout colorimetric detection of catechin was

explored by evaluating the effects of catechin on the color, absorbance value and gray value of the system (Os–Ru nanozyme + TMB + H_2O_2) both in solution system and on paper platform.

Briefly, in the solution system (absorbance measurement), Os–Ru nanozyme (256-fold dilution of the original solution, 20 μL), catechin solution (450 μmol/L, 10 μL) and the reaction substrate solution (200 μL), consisting of TMB (2.0 mM) and H_2O_2 (10 mM), were added in 96-well plate. After reacting at 20°C for 10 min., the photos and the UV absorption spectra of the reaction system at 500~800 nm were taken and measured via iphone 14pro and microplate reader, respectively. The group without adding catechin or Os–Ru nanozyme was set as control groups.

On the paper platform (gray value readout), Os–Ru nanozyme solution (64-fold dilution of the original solution, 10 μL) was firstly added dropwise on clean test paper with diameter of 8 mm, and allowed it to dry naturally at 20°C. Then catechin solution (450 μmol/L, 10 μL) was added dropwise to the test paper and also allowed it to dry naturally at 20°C. Next, reaction substrate solution (20 μL), consisting of TMB (3.75 mM) and H_2O_2 (10 mM), was added dropwise to the test paper and reacted at 20°C for 10 min. After that, the color of the test paper was recorded using iphone 14pro and the gray value (G) of the test paper was obtained by using Image J software to possess the photos. The group without adding catechin or Os–Ru nanozyme was set as control group, and the group without adding catechin and Os–Ru nanozyme was set as blank group. $\triangle$G was the difference between the G value of the blank group and the G value of the test group or control groups.

Optimization of Os–Ru nanozyme sensing conditions

To achieve the best sensing performance of Os–Ru nanozyme for dual-readout colorimetric analysis (absorbance measurement in solution system and gray value readout on paper platform), the sensing conditions in the solution system and on the paper platform were both optimized.

For the solution system (absorbance measurement), the optimization conditions included Os–Ru nanozyme concentration (expressed as dilution ratio of the original solution: 0, 2, 4, 8, 16, 32, 64, 128, 256, 512, 1024, 2048 fold), reaction time (0, 5, 10, 15, 20, 25, 30 min), reaction temperature (20, 25, 30, 35, 40, 45, 50, 55, 60, 65°C), and the pH of reaction system (2, 2.5, 3, 3.5, 4, 4.5, 5, 5.5, 6, 6.5, 7, 7.5, 8, 8.5, 9). Briefly, Os–Ru nanozyme solution at different dilution ratios (20 μL) was mixed with reaction substrate solution (200 μL), which consisted of TMB (2.0 mM) and H_2O_2 (10 mM), in 96-well plate first. Then the above system was reacted under specific pH and temperature conditions for certain reaction time. Subsequently, the absorbance values at 650 nm of the system were measured using microplate reader. The group without adding reaction substrate solution was set as blank control group, and the final absorbance values of all groups were the absorbance values after subtracting the absorbance value of blank control group. Finally, the absorbance values were plotted as the vertical axis, and the Os–Ru nanozymes concentration, reaction temperature, reaction time, and reaction system pH were plotted as the horizontal axis.

As for the paper platform (G value analysis), the optimized conditions included the concentration of Os–Ru nanozyme (expressed as dilution ratio of the original solution: 0, 2, 4, 8, 16, 32, 64, 128, 256, 512, 1024, 2048 fold), reaction time (2, 5, 10, 15, 20, 25 min), reaction system pH (2, 2.5, 3, 3.5, 4, 4.5, 5, 5.5, 6, 7, 8, 9) and the concentration of TMB (4.58 , 3.75, 2.91, 2.08, 1.25, 0.41 mM). In short, the optimization procedure was as follows: Os–Ru nanozyme solution at different dilution ratios (10 μL) was added dropwise to the test paper followed by drying naturally at 20°C. Then, reaction substrate solution (20 μL) consisting of TMB (certain concentration) and H_2O_2 (10 mM) was added dropwise to the test paper. After reacting under 20°C and specific pH for a certain time, the color of the test paper was recorded by taking photo with iphone 14pro and the G value of the test paper was obtained via

processing the photos using image J software. The test paper without adding reaction substrate solution was set as the blank group, and the △G value of the test paper was the difference between the G value of the blank group and the test group. Finally, the ΔG values were plotted as the vertical axis, and the concentration of Os–Ru nanozyme, reaction time, reaction system pH and the concentration of TMB were plotted as the horizontal axis.

Storage stability of the paper colorimetric platform

The storage stability of the paper platform at room temperature **(**20°C**)** was evaluated by measuring the catalytic reaction activity of the test paper containing Os–Ru nanozyme on days 0, 10, 20, 30, 40, 50, and 60. Briefly, the reaction substrate solution (20 μL) consisting TMB (3.75 mM) and H_2O_2 (10 mM), was added dropwise to the test paper (containing Os–Ru nanozyme) with different storage days. Then, the test paper was reacted at 20°C for 10 min. Next, the color of the test paper was recorded by capturing images using a device consisted of an iPhone 14 Pro and a small LED photography studio (**Fig. S1**), and the G value of the test paper was obtained via transforming the color of the photo using the Image J software. The G value on day 0 was set as 100% relative activity.

Results and discussion

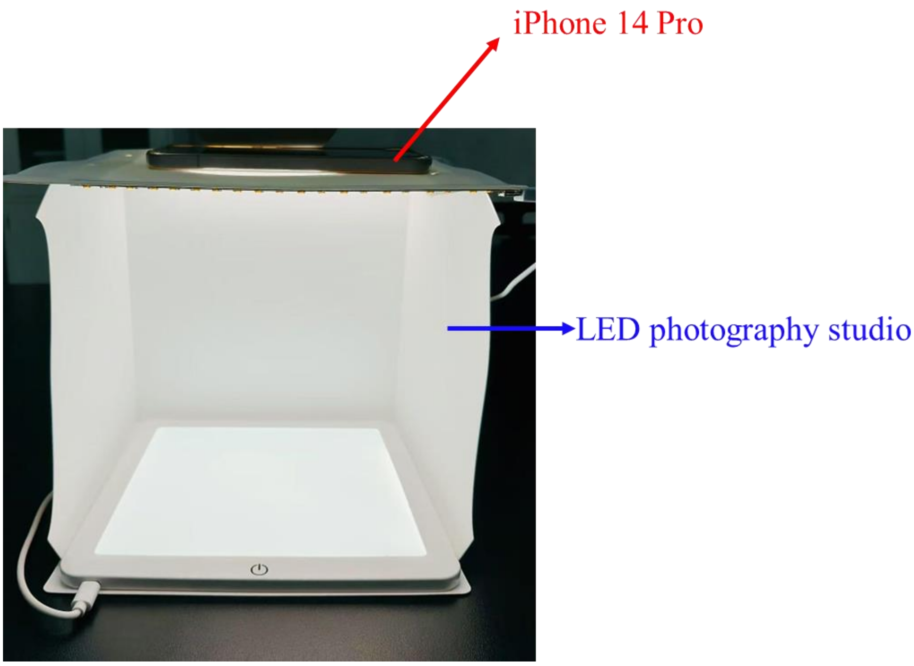

Fig. S1. Schematic of the device used for paper platform image capture.

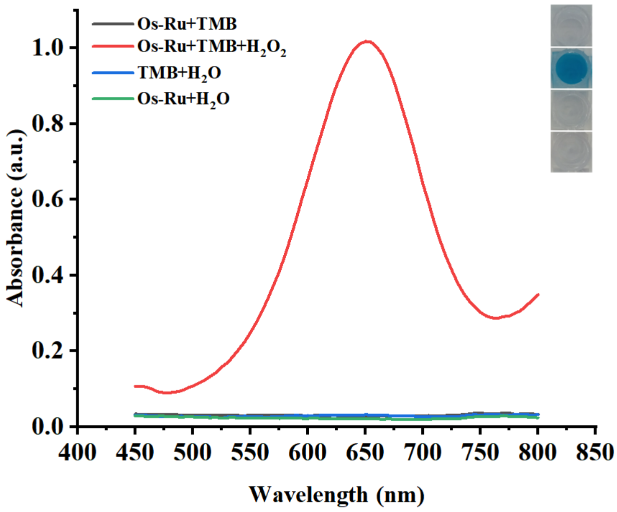

Fig. S2. The peroxidase-mimicking activity of Os–Ru nanozyme (the UV-vis absorption spectra measured at 500 ~ 800 nm after 10 min of reaction).

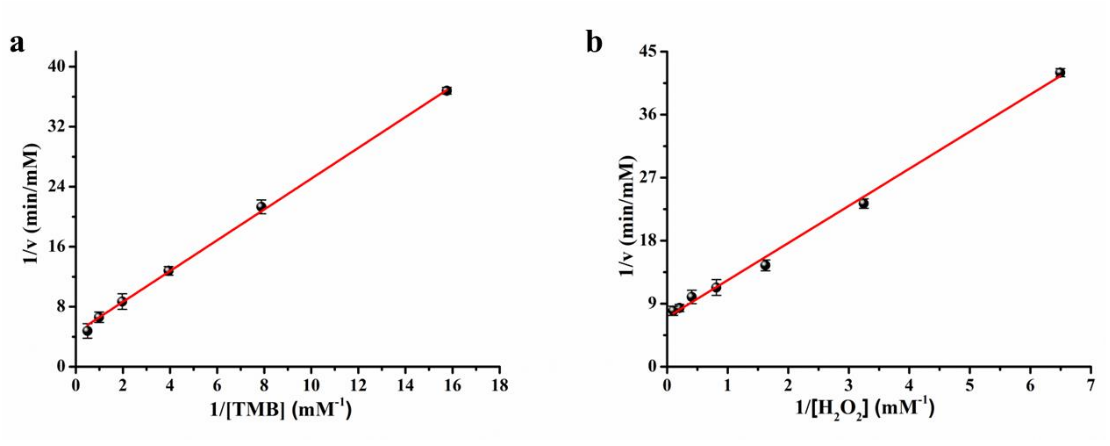

Fig. S3. Steady-state kinetic assay results of Os–Ru nanozyme. (a) Lineweaver-Burk plot with TMB as substrate and (b) Lineweaver-Burk plot with H_2O_2 as substrate.

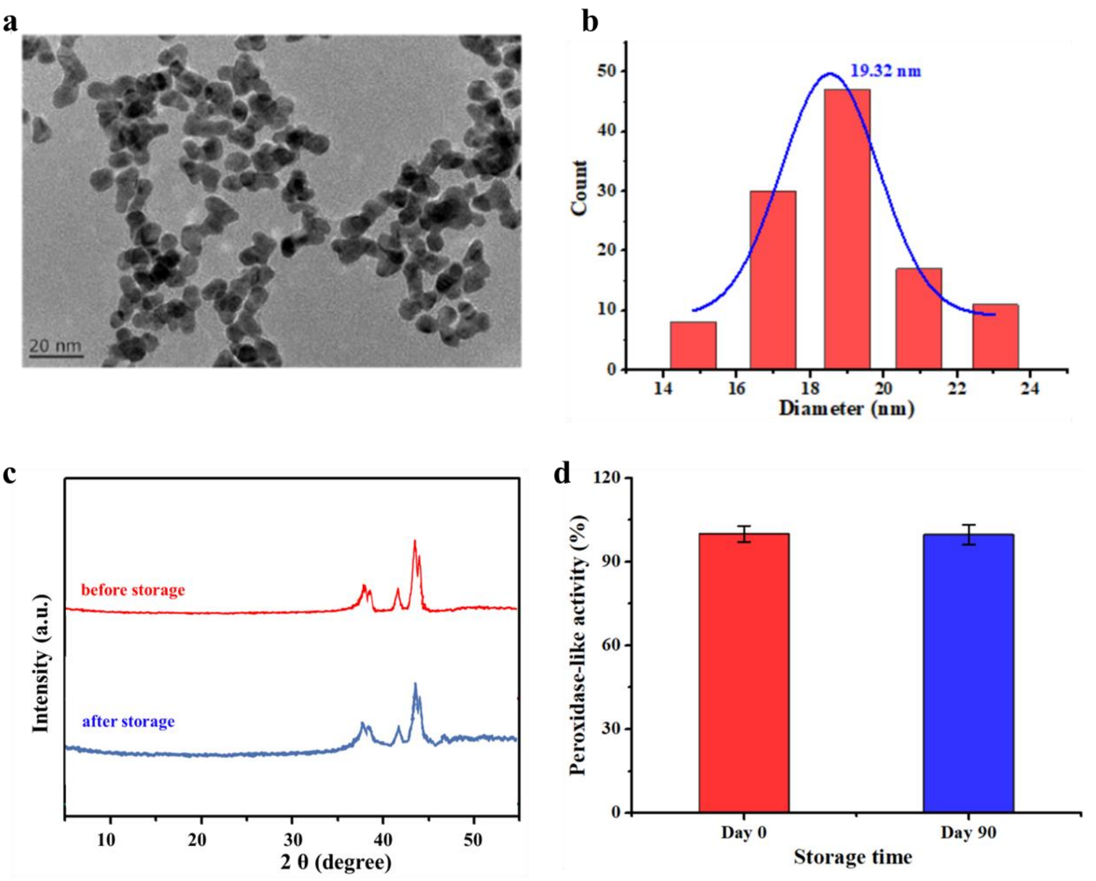

Fig. S4. (a) TEM image, (b) size distribution, (c) XRD patterns and (d) peroxidase-mimicking activity of Os–Ru nanozyme after storage for 90 days.

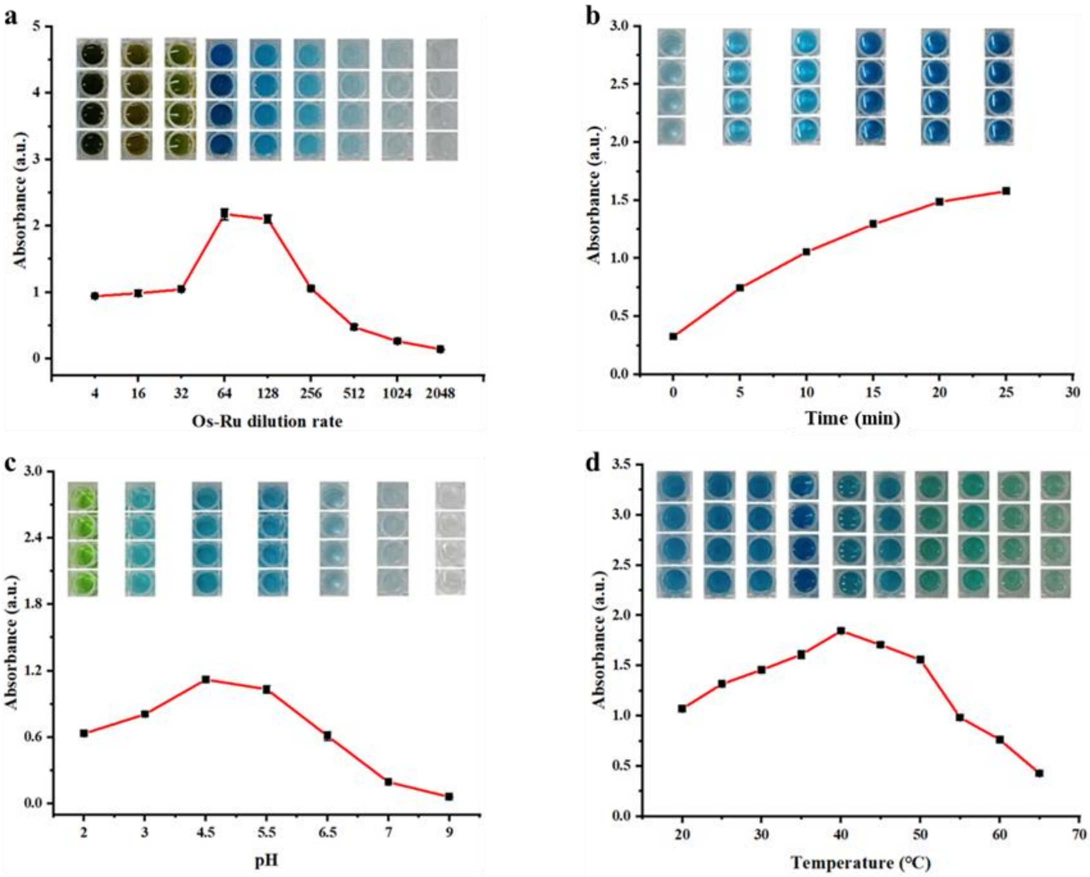

Fig. S5. Optimization of Os–Ru nanozyme sensing conditions in solution system, including (a) the concentration of Os–Ru nanozyme, (b) rection time, (c) reaction pH and (d) reaction temperature.

Fig. S5a showed that the absorbance values of the system gradually increased and then decreased with the increase of the dilution ratio (decrease of Os–Ru nanozyme concentration). The absorbance value of the system reached the maximum (about 2.18) when the Os–Ru nanozyme solution is diluted 64 times. When the dilution ratio was less than 64, the colors of the reaction system would become yellow green, brown or even black, and the absorbance values of the system decreased instead. This phenomenon may be due to the excessive oxidation of TMB caused by the excess of Os–Ru nanozyme in the system. While the dilution ratio was greater than 64, the colors of the reaction system gradually became lighter from blue, which corresponded to the gradual decrease of the absorbance value of the system. When the Os–Ru nanozyme solution was diluted to 256 times, the color of the system was blue and the absorbance

value was within the optimal range (0.8 ~ 1.2), so 256 was chosen as the optimal dilution ratio.

As shown in **Fig. S5b**, it was found that the absorbance values of the reaction system gradually increased and the blue color of the system gradually became darker with the increase of the reaction time. While at the reaction time of 10 min, the absorbance value of the system was around 1.0, so 10 min was chosen as the optimal reaction time.

From **Fig. S5c**, it could be seen that with the increase of system pH, the absorbance values of the reaction system increased and then decreased, and the color of the system showed the change of bright green - light blue green – blue - light blue - colorless. The above phenomenon may be attributed to the fact that TMB undergone secondary oxidation under excessively acidic conditions, whereas Os–Ru nanozyme could not exhibit peroxidase-mimicking activity under alkaline conditions.[2] At pH 4.5, the color of the system is blue and the absorbance value at 650 nm reached the maximum (about 1.12). Therefore, pH 4.5 was selected as the optimal reaction system pH.

Fig. S5d showed that as the temperature increases, the absorbance value of the reaction system tended to increase and then decrease, and the color of the system showed a blue - dark blue - blue green -green - yellow change. This may be due to the fact that the activity of Os–Ru nanozyme was enhanced with the increase of reaction temperature, which could better catalyze the oxidation of TMB, making the system blue darker and the absorbance value increased. While the temperature was too high, most of the TMB in the system may undergo secondary oxidation,[2] which leaded to a change in the color of the system towards green and even yellow, accompanied by a decrease in the absorbance value. When the reaction temperature was 20°C, the absorbance value of the system was around 1.0 and with blue color, so 20°C was chosen as the optimal reaction temperature.

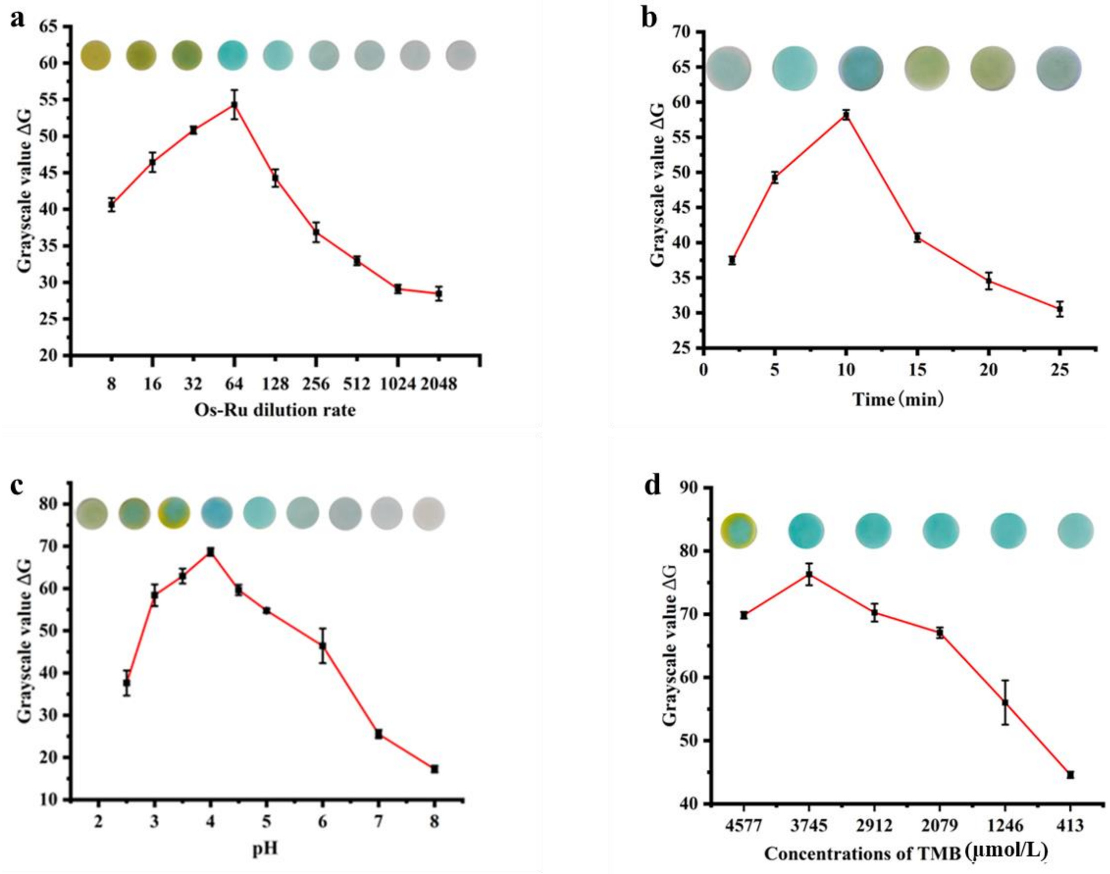

Fig. S6. Optimization of Os–Ru nanozyme sensing conditions on paper platform, including (a) the concentration of Os–Ru nanozyme, (b) rection time (c) pH and (d) the concentration of TMB.

Fig. S6a displayed that with the increase of the dilution ratio (decrease of Os–Ru nanozyme concentration), the △G value of the paper first progressively increased and then leveled off, and the color of the paper gradually changed from yellow to yellow-green to blue to light blue and finally to colorless. When the dilution ratio was 64, the ΔG value of the paper reached the maximum (54.30) and was accompanied by the most obvious blue color of the paper. When the dilution ratio was less than 64, the paper color would turn into yellow or even yellow-green, with the ΔG value of decreased, which was due to the excessive oxidation of TMB caused by the excessive amount of Os–Ru nanozyme in the system. While the dilution ratio was more than 64, the ΔG value decreased significantly with the paper color became light blue or even colorless, which could be attributed to the catalytic activity of Os–Ru

nanozyme was weakened by the low concentration. Thus, 64 was chosen as the optimal dilution ratio.

Fig. S6b showed that with the extension of the reaction time, the ΔG value of the paper first increased and then decreased, and the paper color gradually changed from light blue to dark blue to yellow-green and finally to nearly colorless. When the reaction time was 10 min, the ΔG value reached the maximum (about 58.20), and the blue color of the paper was most obvious at this time. When the reaction time was greater than 10 min, the paper color changed from dark blue to yellow-green and finally to nearly colorless, accompanied by a decrease in the ΔG value of the paper. This may be due to the long reaction time leading to the secondary oxidation of TMB as well as the decomposition of oxTMB.[3] Thence, 10 min was selected as the optimal reaction time.

From the result of **Fig. S6c**, it could be found that the pH possessed a large influence on the activity of Os–Ru nanozyme. As the pH increased, the ΔG value of the paper first gradually increased and then decreased, with the color of paper changed from yellow - yellow green - blue - light blue - colorless. This phenomenon also may be due to TMB undergone secondary oxidation under excessively acidic conditions, and Os–Ru nanozyme could not exhibit peroxidase-mimicking activity under alkaline conditions.[2] The highest ΔG value (about 68.64) of paper was found at the pH of 4.0, and the paper color was blue at this time, so pH = 4.0 was chosen as the optimal reaction pH.

It could be found from **Fig. S6d** that the concentration of TMB solution also had a large impact on the color and grey value of paper. As the concentration of TMB solution decreased, the ΔG value of the paper increased and then decreased, and the paper color changed from blue-yellow to blue and then to light blue. When the concentration of TMB solution was 3.75 mM, the ΔG value of the paper was the highest and the color was the bluest at that time, so 3.75 mM was selected as the best concentration of TMB solution.

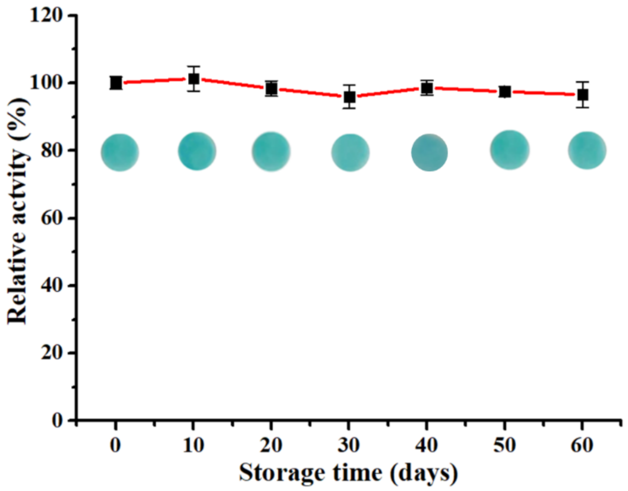

Fig. S7. Storage stability of the Os–Ru nanozyme-based paper platform.

Table S1. Recovery efficiency of the catechin spiked in real samples

Method	Samples[a]	Added (μmol/L)	Detected (μmol/L)	Recovery (%)	RSD (%)
**Solution system**	Master Kong	200	195.64 ± 5.88	97.82 ± 2.94	3.01
		400	394.52 ± 26.60	98.63 ± 6.65	6.74
	Vita	200	189.38 ± 8.06	94.69 ± 4.03	4.26
		400	373.08 ± 8.72	93.27 ± 2.18	2.34
	Yulu	200	206.02 ± 6.76	103.01 ± 3.38	3.28
		400	401.44 ± 8.72	100.36 ± 2.18	2.17
**Paper platform**	Master Kong	200	192.34 ± 4.86	96.17 ± 2.43	2.53
		400	383.02 ± 17.52	95.76 ± 4.38	4.57
	Vita	200	183.56 ± 7.02	91.78 ± 3.51	3.82
		400	369.16 ± 11.40	92.29 ± 2.85	3.09
	Yulu	200	199.32 ± 3.22	99.66 ± 1.61	1.62
		400	403.04 ± 11.16	100.76 ± 2.79	2.77
**HPLC**	Master Kong	200	195.80 ± 3.30	97.90 ± 1.65	1.69
		400	393.08 ± 8.04	98.27 ± 2.01	2.05
	Vita	200	189.96 ± 5.64	94.98 ± 2.82	2.97
		400	377.44 ± 7.04	94.36 ± 1.76	1.86
	Yulu	200	205.66 ± 6.12	102.83 ± 3.06	2.98
		400	404.12 ± 9.72	101.03 ± 2.43	2.41

[a] Three green tea beverages were purchased from the local market. The measurement for each sample was performed three times (n = 3).

Table S2. Determination of catechin content in green tea beverages through the dual-readout colorimetric analysis method

**Green tea beverages**	**Catechin content (μmol/L)**	
	Solution system	Paper platform
Master Kong	2208.96	2178.68

Reference

1 A. Rodriguez-Abetxuko, P. Muñumer, M. Okuda, J. Calvo, M. Knez and A. Beloqui, *Adv. Funct. Mater.*, 2020, **30**, 2002990.

2 C. Zhu, H. Yang, X. Cao, Q. Hong, Y. Xu, K. Wang, Y. Shen, S. Liu and Y. Zhang, *Anal. Chem.*, 2023, **95**, 16407-16417.

3 Z. Chen, H. Yao, J. Wang, J. Zhang, T. Zhang, Z. Li, J. Qiao, S. Xiu, X. Hao and J. Hou, *Energy Environ. Sci.*, 2023, **16**, 2637-2645.
